# Effect and Safety of Pioglitazone-Metformin Tablets in the Treatment of Newly Diagnosed Type 2 Diabetes Patients with Nonalcoholic Fatty Liver Disease in Shaanxi Province: A Randomized, Double-Blinded, Double-Simulated Multicenter Study

**DOI:** 10.1155/2023/2044090

**Published:** 2023-06-01

**Authors:** Fu Jianfang, Xiao Wanxia, Gao Xiling, Xu Jing, Yang Wenjuan, Liu Jianrong, He Qingzhen, Ma Kaiyan, Lian Jingxuan, Chen Taixiong, Xu Qian, Li Mengying, Ming Jie, Ji Qiuhe

**Affiliations:** ^1^Air Force Medical University Xijing Hospital, Xi'an, Shaanxi, China; ^2^Genertec Universal Xian Aero-Engine Hospital, Xi'an, Shaanxi, China; ^3^Yan'an People's Hospital, Yan'an, Shaanxi, China; ^4^Xi'an Jiaotong University Second Affiliated Hospital, Xi'an, Shaanxi, China; ^5^Shaanxi Aerospace Hospital, Xi'an Da'xin Hospital, Xi'an, Shaanxi, China; ^6^Chang'an Hospital, Xi'an, Shaanxi, China; ^7^Gaoxin Hospital, Xi'an, Shaanxi, China; ^8^Shangluo Central Hospital, Shangluo, Shaanxi, China

## Abstract

**Objective:**

The aim of study was to evaluate the effect and safety of pioglitazone-metformin combined treatment in the newly diagnosed type 2 diabetes patients with nonalcoholic fatty liver disease.

**Methods:**

A total of 120 newly diagnosed type 2 diabetes patients with nonalcoholic fatty liver disease from 8 centers were randomly divided into the control group (metformin hydrochloride) and the test group (pioglitazone hydrochloride and metformin hydrochloride).

**Results:**

Compared to the control group, after treatment, the proportion of people with mild and moderate fatty liver increased, and the proportion of people with severe fatty liver decreased, and this change was more obvious in the population with moderate and severe fatty liver. The level of *γ*-GT decreased in both groups before and after treatment, which was statistically significant, and there was also a statistically significant difference in the level of *γ*-GT between the two groups after 24 weeks. There were no significant statistically differences in blood lipid, body weight, and waist circumference between the test group and the control group. Logistic regression analysis found that BMI is one of the risk factors for fatty liver. There was also no significant difference in the incidence of serious adverse events between the two groups (control group: 10.00% and test group: 6.67%, *P* = 0.74).

**Conclusion:**

Combined treatment with pioglitazone-metformin can effectively reduce liver fat content and gamma-GT level in newly diagnosed diabetic patients with nonalcoholic fatty liver disease, and adverse events do not increase compared with the control group, showing good safety and tolerance. This trial is registered with ClinicalTrials.gov NCT03796975.

## 1. Introduction

As one of the most common chronic diseases, the prevalence rate of diabetes in China has skyrocketed, and the estimated prevalence of diabetes in Chinese adults is 11.6%, with type 2 diabetes predominating [1]. Type 2 diabetes mellitus (T2DM) is a metabolic disease characterized by hyperglycemia, which is caused by insulin secretion deficiency or/and insulin resistance (IR) caused by a combination of genetic and environmental factors [2]. NAFLD is a type of metabolic stress-induced liver injury, which is closely related to insulin resistance and genetic susceptibility. The spectrum of the disease includes nonalcoholic simple fatty liver (NAFL) [3, 4]. The incidence of NAFLD in patients with T2DM in China is as high as 49-62% [5]. Studies have shown that T2DM increases the NAFLD risk by 36.7 times [6]. The prevalence rate of adult NAFLD in China has reached 29.2% [5]. Our team's study showed that the risk of T2DM in NAFLD increased by 4.46 times and the risk of prediabetes increased by 1.64 times [7]. Current studies have confirmed that IR may be the common pathogenesis of type 2 diabetes and nonalcoholic fatty liver, and IR may be the initiating factor and key factor in the onset of NAFLD [8].

Clinically, NAFLD is currently treated with multiple drugs, one of which is insulin sensitizer. Many literature reports that insulin sensitizers can effectively improve NAFLD [9–12]. At present, high-quality studies have found that pioglitazone can better improve liver enzyme levels and insulin resistance in patients with NAFLD. In addition, pioglitazone can also improve liver histological performance in patients, but there is an unfavorable risk of weight gain, which limits its clinical use, and metformin can improve liver enzyme levels and reduce weight in patients with NAFLD [13, 14]. Although pioglitazone or metformin has more clinical evidence-based medical evidence for the treatment of nonalcoholic fatty liver disease, the combination of pioglitazone and metformin or its compound preparation has very limited research and reports on the treatment of nonalcoholic fatty liver.

Therefore, the purpose of this study is to observe the therapeutic effect of pioglitazone-metformin combined treatment in type 2 diabetes patients with NAFLD. While using pioglitazone to improve the patient's liver histological performance, additional metformin can offset the risk of weight gain caused by pioglitazone. In addition, both can improve the patient's liver enzyme levels and insulin resistance, thereby increasing patient compliance, and bring obvious benefits to patients with type 2 diabetes and fatty liver.

The hypoglycemic effect of pioglitazone hydrochloride and metformin hydrochloride tablets (15 mg pioglitazone/500 mg metformin) has been fully verified and affirmed. In this study, ultrasound liver fat content and liver enzyme levels were used as primary endpoint to evaluate the efficacy of NAFLD. In this multicenter clinical trial, we used a randomized, double-blind, double-simulated method, with metformin as the control group and ultrasound liver fat content and liver enzyme levels as the primary endpoints to evaluate the effect of combined treatment of metformin and pioglitazone in type 2 diabetes patients with NAFLD; the risk of adverse events is used to evaluate the safety of combined treatment of metformin and pioglitazone.

## 2. Materials and Methods

### 2.1. Study Design and Participants

This study was a randomized, double-blind, double-simulation, and positive drug control multicenter clinical trial (ClinicalTrials.gov registration number: NCT03796975). The experimental protocol was approved by the Medical Ethics Committee of Xijing Hospital, Air Force Military Medical University, and completed jointly by 8 hospitals.

Inclusion criteria are as follows: according to WHO criteria, age 18-70 years old, BMI between 21 and 35 kg/m^2^, and HbA1c level between 7.0 and 10.0% newly diagnosed type 2 diabetes mellitus patients with nonalcoholic fatty liver disease. In addition, the patient had never received oral hypoglycemic drugs or insulin and had not participated in any drug trials within 3 months prior to enrollment. Exclusion criteria included uncontrolled hyperglycemia at screening (fasting blood glucose (FBG) ≥ 240 mg/dL), liver disease other than NAFLD such as chronic viral hepatitis (B or C), alcoholism, hemochromatosis, alpha-1 antitrypsin deficiency, autoimmune hepatitis, Wilson's disease, primary sclerosing cholangitis, or primary biliary cirrhosis or any causes of cirrhosis were also excluded; previous thiazolidinedione treatment, use of immunomodulatory, antiobesity, PCSK9 inhibitors, and use of anti-NASH drugs (vitamin E, ursodeoxycholic acid, S-adenosylmethionine, betaine, silymarin, gemfibrozil, anti-TNF therapies, and probiotics) in 3 months prior to randomization were excluded. In addition, smoking (not smoking < 1 year), drinking (male > 30 g/d and female > 20 g/d), and drug abuse or psychiatric disease were also excluded. All participants provided written informed consent.

### 2.2. Randomization and Masking

Through a computer-generated centralized management program, all participants were randomized by a stratified computed randomization procedure to account for age, sex, and BMI to test group or control group at a ratio of 1 : 1 and were masked to the treatment assignment. The test group received pioglitazone-metformin combined treatment, and the control group received metformin treatment and matching placebo treatment. The electronic master randomization list was only accessible to the assigned randomization list managers, and study sites received sealed opaque envelopes for unblinding in cases of emergency. Enrollment was performed at the respective site. Randomization and assignment to the double-blind study drug were done by central pharmacy personnel, who had access to the computer-generated randomization scheme. The appearance, color, and smell of all simulated tablets are the same as the corresponding drugs. Liver fat content testing in all test centers is performed by fixed personnel, and all testing personnel have been uniformly trained to ensure the same measurement quality.

### 2.3. Procedures

In order to improve gastrointestinal tolerance, all patients in the group received a weekly dose titration. The initial dose of the test group was once a day, 500 mg metformin and 15 mg pioglitazone (both from Hangzhou Zhongmei Huadong Pharma Ceutical Co., Ltd.) were taken before breakfast or dinner, and after 1 week of treatment, the dose was adjusted to twice a day, before breakfast and dinner. The initial dose of the control group was 500 mg metformin, once a day, before breakfast or dinner, and after 1 week of treatment, the dose was adjusted to twice a day, before breakfast and dinner. Patients were visited at 2, 4, 8, 12, 16, 20, and 24 weekends after treatment to evaluate the effectiveness and safety of the trial drug.

Participants will withdraw from the experiment when the following situations occur: severe hypoglycemia requires the assistance of others; fasting blood glucose < 2.8 mmol/L or two random blood glucose < 3.9 mmol/L caused by overdose; in the absence of other concomitant diseases, frequent urination, thirst, weight loss, or other aggravation due to high blood sugar; and asymptomatic FPG confirmed by two measurements > 13.3 mmol/L.

### 2.4. Quantitative Ultrasound for Liver Steatosis

In our study, we mainly used controlled attenuation parameter (CAP) for liver fat quantification, which is best studied and clinically available technique for liver fat quantification, with the first clinical studies dating back to 2010 [15]. The CAP is measured at the center frequency of the probe from the ultrasound data and correlates with the degree of ultrasound attenuation caused by intrahepatic fat accumulation [16]. The CAP demonstrated excellent diagnostic accuracy in the detection of S1, S2, and S3 hepatic steatosis by liver biopsy. Several studies, in which liver biopsy was used as the reference standard, have reported a good performance of the CAP in grading liver steatosis [17, 18].

### 2.5. Outcomes

The primary end points are liver fat content (ultrasonic quantitative determination of liver fat content), ALT, AST, gamma-GT levels, and insulin resistance index. The secondary endpoints are HbA1c, intravenous fasting blood glucose, 2 h postprandial blood glucose, total cholesterol, triglyceride, HDL cholesterol, LDL cholesterol, waist circumference, and body weight. Safety indicators such as medication compliance, vital signs, hypoglycemic events, nonhypoglycemic events, blood routine, urine routine, and electrocardiogram were analyzed.

### 2.6. Statistical Analysis

The statistical analysis was performed using SAS (9.1.3) statistical analysis software. The *t*-test (normal data) or Wilcoxon rank-sum test (nonnormal data) was used to test the balance between groups, basic indicators, and efficacy results. All the included cases enter the complete set analysis, and all the included cases enter the safety analysis. The incidence of adverse events was compared between the two groups by *χ*^2^ test. *P* < 0.05 was used as the standard for statistical significance test in this study.

## 3. Results

### 3.1. Baseline Characteristics

**Figure 1:**
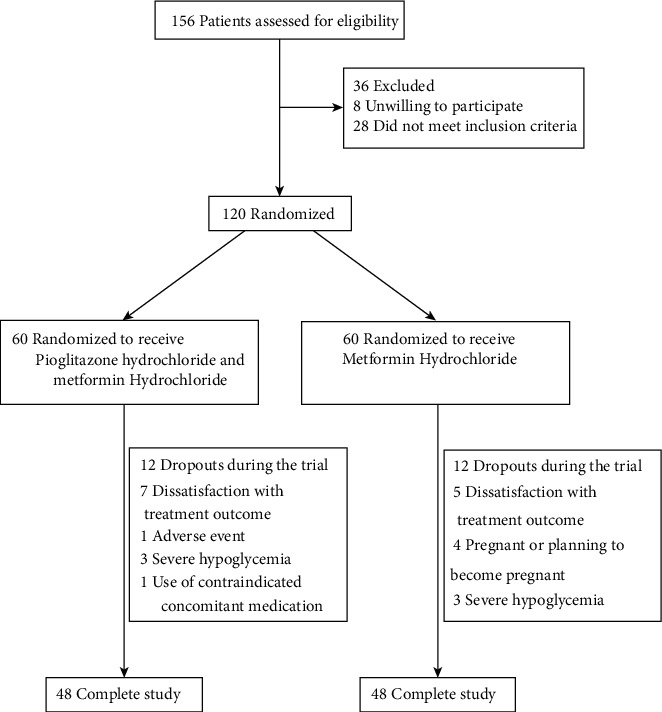
Consolidated Standards of Reporting Trials (CONSORT) diagram of participant flow through the study.

This study enrolled a total of 120 type 2 diabetes patients with NAFLD from 8 hospitals in Shaanxi Province. As shown in the Consolidated Standards of Reporting Trials (CONSORT) diagram in Figure 1, all patients received their assigned treatment; in total, 96 participants completed the study (control group: 48 cases and test group: 48 cases), and 12 patients in each treatment group withdrew from treatment. Among them, 12 cases were unsatisfactory with the treatment effect, 6 cases of severe hypoglycemia, 1 case of adverse events, 1 case of using contraindicated concomitant drugs, and 4 cases withdrew from the study because of planning pregnancy. There were 96 cases that meet the requirements (control group: 48 cases and test group: 48 cases). The baseline demographic, clinical, laboratory, and basic characteristics of the two groups were similar, except for differences in waist-to-hip ratio between the two groups (Table 1). Compliance was high (96%) and similar in each treatment group.

### 3.2. Primary Endpoint Measurement

**Figure 2:**
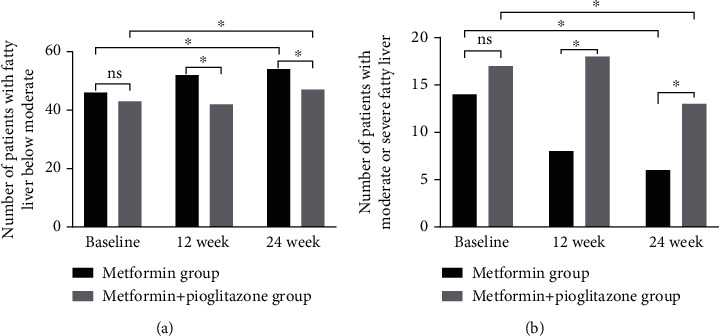
(a) The number of patients with mild fatty liver at baseline, 12 weeks, and 24 weeks. (b) The number of patients with moderate and severe fatty liver at baseline, 12 weeks, and 24 weeks. ^∗^*P* < 0.05.

After 24 weeks of treatment, 8 (13%) of the 60 patients in the test group had substantial improvement in degree of liver fat content, and the degree of liver fat content decreased from moderate and severe grade to mild grade. In contrast, 4 of 60 patients (6%) in the metformin group saw their liver fat content drop from moderate or severe to mild. Statistical analysis showed that the differences in the test group before and after treatment and compared with the metformin group were statistically significant. The number of patients with moderate or lower liver fat content in the pioglitazone-metformin combined treatment group increased from 46 (76.7%) to 54 (90.0%) and the metformin group increased from 43 (71.7%) to 47 (78.3%). The changes before and after treatment in the pioglitazone-metformin combined treatment group were statistically significant (*P* < 0.05), but there was no statistical difference compared with the metformin group (*P* > 0.05) (Figure 2). In patients with severe fatty liver, both groups could improve liver fat content after treatment. However, compared with the metformin group, the pioglitazone-metformin combined treatment group could significantly improve the liver fat content in patients with severe fatty liver; especially at 24 weeks, the difference was more significant.

**Figure 3:**
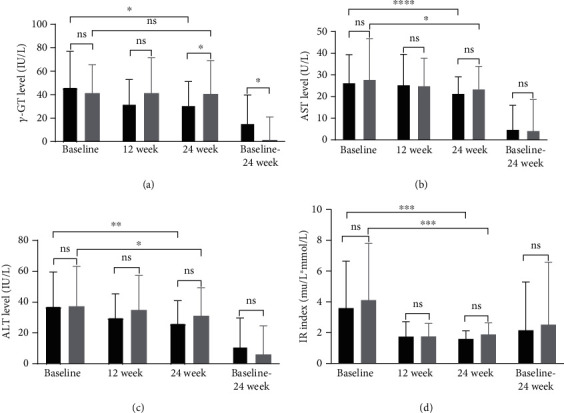
(a) Plasma *γ*-GT level at baseline, 12 weeks, and 24 weeks and changes in *γ*-GT level from baseline to 24 weeks. (b) Plasma AST level at baseline, 12 weeks, and 24 weeks and changes in AST level from baseline to 24 weeks. (c) Plasma ALT level at baseline, 12 weeks, and 24 weeks and changes in ALT level from baseline to 24 weeks. (d) IR index level at baseline, 12 weeks, and 24 weeks and changes from baseline to 24 weeks. AST: aspartate aminotransferase; ALT: alanine aminotransferase; IR: insulin resistance index; ns: no significance. I bars and T bars denote standard deviations. ^∗^*P* < 0.05, ^∗∗^*P* < 0.01, ^∗∗∗^*P* < 0.00, and ^∗∗∗∗^*P* < 0.0001.

Compared with the metformin group, pioglitazone-metformin combined treatment was associated with a significant decrease in *γ*-GT in T2DM patients. After 24 weeks of treatment, the *γ*-GT level in the test group was significantly reduced, while the metformin group had no significant changes. Statistical analysis showed that the level changes in the test group before and after treatment or compared with the metformin group were statistically different. In terms of liver enzyme levels, compared with the metformin group, after 12 weeks and 24 weeks of treatment, there was no significant difference in AST and ALT levels in pioglitazone-metformin combined treatment group, but there were significant statistical differences between the two groups before and after treatment. The same is true for the insulin resistance index compared with the metformin group. The IR index of patients treated with the combination of pioglitazone and metformin did not improve significantly, but the HOMA-IR changes before and after treatment in the two groups were statistically significant (Figures 3(a)–3(d)).

### 3.3. Secondary Endpoint Measurement

After 24 weeks of treatment, the HbA1c level of the pioglitazone-metformin combined treatment group and the metformin treatment group decreased significantly (pioglitazone-metformin combined treatment: 1.78% (95% CI: 1.53-2.04) and metformin treatment: 1.71% (95% CI: 1.46-1.96)), but there was no significant difference between the two groups (Table 2). After 24 weeks of treatment, the FPG of the pioglitazone-metformin combined treatment group decreased by 1.48 mmol/L (95% CI: 0.85-2.11), and the metformin group FPG decreased by 1.58 mmol/L (95% CI: 0.98-2.19). There was no significant statistical difference between the pioglitazone-metformin combined treatment group and the metformin group. However, the differences between the two groups before and after treatment were statistically significant (Table 2). Compared with the metformin group, there was no statistically significant reduction in PBG after 24 weeks of combined treatment in the pioglitazone and metformin group. However, compared with before treatment, the PBG level of the pioglitazone-metformin combination treatment group decreased significantly (4.00 (95% CI: -7.50-12.10)), while there was no significant difference in the metformin group (Table 2). There is no statistically significant difference in concentrations of TC, TG, and LDL-C, and HDL-C was observed in the pioglitazone-metformin combined treatment group after 24-week treatment compared to the metformin group. Moreover, there was also no significant change in the pioglitazone-metformin combination treatment group and the metformin group compared with the baseline level (Table 2).

There is no statistically significant reduction in BMI, and waist circumference was observed in the pioglitazone-metformin combined treatment group after 24-week treatment compared to the metformin. Moreover, there was no significant change in the pioglitazone-metformin combined treatment group and the metformin group when compared with the baseline level (Table 2).

### 3.4. Safety Evaluation

In this study, the compliance of pioglitazone-metformin combined treatment group was 95.79%, and that of the metformin group was 95.10%. As shown in Table 3, there was no statistical difference in the risk of adverse events and treatment-related adverse events between the two groups. The specific adverse events in each group are shown in Table 4; there were 7 adverse events in the pioglitazone-metformin combined treatment group, 5 of which were related to treatment, including 3 patients of mild diarrhea, 1 patient of urine occult blood, and 1 patient of mild cardiac insufficiency; a total of 14 adverse events occurred in the metformin group, 10 of which were related to treatment, and all of which were mild diarrhea. One serious adverse event occurred in both groups, one patient in the pioglitazone-metformin combined treatment group had mild hypertension, and one patient in the metformin group had moderate angina; both were considered to be irrelevant to treatment. There were no patients of withdrawal due to adverse events in each group.

## 4. Discussion

Nonalcoholic fatty liver disease is a metabolic stress liver injury closely related to insulin resistance and genetic susceptibility. Except that the patient has no history of excessive drinking, its pathological changes are similar to alcoholic liver disease (ALD). The disease spectrum includes nonalcoholic fatty liver (NAFL), nonalcoholic steatohepatitis (NASH), and related liver cirrhosis and hepatocellular carcinoma [3, 4]. The harm of NAFLD is not limited to the high morbidity and mortality associated with liver disease. Increasing evidence shows that NAFLD is a metabolic disorder-related multisystem disease, which is more common in obesity or T2DM patients and has similar adverse outcomes with these two diseases; in addition, patients with NAFLD are twice as likely to die from cardiovascular disease as from liver disease [19]. Studies have found that the incidence of T2DM or fasting blood glucose impairment in NAFLD patients is as high as 18-33% [20]. Our team's research found that NAFLD is an important predictor of diabetes. Studies have shown that the all-cause mortality of individuals with NAFLD is higher than that of the general population [21], making it an important health problem. Despite the heavy burden imposed by NAFLD, there is still no effective treatment strategy [22, 23].

In this double-blind, randomized, placebo-controlled trial, pioglitazone-metformin combined treatment met the predefined primary endpoint and can significantly reduce the liver fat content of newly diagnosed type 2 diabetes with moderate or severe fatty liver patients. In addition, after 24 weeks of treatment, the patient's *γ*-GT level also decreased, although longer-term outcome studies are needed to confirm this result. Pioglitazone has been shown to improve histological inflammation and/or fibrosis compared to placebo in several moderate-sized randomized controlled trials [24]. The PIVENS trial compared 30 mg pioglitazone or vitamin E to placebo over 96 weeks and found no improvement in NASH but did demonstrate improvements in lobular inflammation, steatosis, and liver biochemistry [24]. Other studies have suggested that the beneficial effects of pioglitazone may be related to its insulin-sensitizing properties, which reduce insulin resistance in the adipose tissue, muscle, and liver [25]. A meta-analysis of 392 patients from 5 RCTs confirmed these findings in diabetic and nondiabetic patients, showing that pioglitazone resolved NASH (odds ratio 3.51, 95% confidence interval (CI) 1.76-7.01) and improved advanced fibrosis (odds ratio 4.53, CI 1.52–13.52) [26]. These findings were confirmed by a series of recent meta-analyses which have also shown that pioglitazone can significantly improve liver fibrosis and steatosis in NASH [27, 28]. In addition, metformin's benefits, including inhibiting hepatic gluconeogenesis, modifying hepatic fatty acid metabolism, increasing fatty acid oxidation, reducing lipogenesis, enhancing insulin sensitivity, and increasing antioxidant properties, are well-established [29]. Therefore, we believe that pioglitazone-metformin combination therapy is safe and well tolerated regardless of the severity of the underlying disease.

No statistically significant changes were observed in patients with mild liver content; this is consistent with previous studies showing that insulin sensitizers can effectively improve NAFLD [9–13]. Although the pioglitazone-metformin combined treatment and metformin alone have no significant differences in biological indicators such as HbA1c, fasting blood glucose, 2 h postprandial blood glucose, and insulin resistance, this is consistent with the results of previous studies, and the two groups have the same risk of increasing waist circumference and BMI. It shows that even after adding pioglitazone, the risk of weight gain in patients does not increase significantly, indicating that metformin can significantly offset the risk of weight gain caused by the use of pioglitazone.

In terms of safety, the addition of metformin did not significantly increase the risk of adverse events. The pioglitazone-metformin combination group had the same risk of adverse events as the metformin group, and they were well tolerated. Drug-related adverse reactions were mainly diarrhea, occult blood in the urine, and insufficient blood supply to the myocardium. These adverse events were, however, mainly transient and mild-to-moderate severity. A total of 2 serious adverse events occurred in the two groups, including hypertension and angina pectoris, with mild-to-moderate severity. Both cases were considered to be unrelated to the study drug. However, whether pioglitazone affects the cardiovascular system and the occurrence of adverse cardiovascular events remains to be confirmed by further increasing the number of studies. NAFLD is a recent research hotspot, and there is still no specific guideline for the treatment plan of NAFLD patients. Although many hypoglycemic drugs have been tested in NAFLD patients, there are currently no approved drugs for the treatment of NAFLD [30].

## 5. Limitation

Although our research is a randomized, double-blinded, double-simulated multicenter study, but our research still has some limitations. First of all, our study did not incorporate the gold standard of liver biopsy into our main results because of the expected early stages of NAFLD in these patients and the short duration of intervention, which weakened the reliability of our results to a certain extent. Moreover, this study did not use multiple imputation to account for missing values but performed maximum likelihood methods for the primary end point. In addition, 24 patients were lost during follow-up (20%) in our study. This could be a huge missing and may represent a bias to the study. Finally, due to our inclusion of patients and the limited duration of intervention, long-term, large-scale placebo-controlled, rigorous randomized controlled studies are still required to confirm our results.

## 6. Conclusion

In conclusion, the results of this study showed that in the observation period of 24 weeks, in newly diagnosed type 2 diabetes patients with NAFLD, the pioglitazone-metformin combined treatment can effectively reduce liver fat content, especially in patients with moderate to severe fatty liver and *γ*-GT levels without increasing body weight and waist circumference, and the risk of adverse events is not significantly increased compared with metformin alone. Therefore, for this group of patients, pioglitazone-metformin combined treatment for initial treatment may have double benefits in the improvement of hypoglycemic and liver fat, which still needs to be supported by larger sample size studies.

## Figures and Tables

**Table 1:** Baseline characteristics of study population.

	Metformin + pioglitazone (*n* = 60)	Metformin (*n* = 60)	*P* ^∗^
Demographics			
Age	57.63 (13.44)	61.25 (11.93)	0.8351
Gender			
Male	41 (68.33)	45 (75.00)	0.4178
Female	19 (31.67)	15 (25.00)	
Physical examination			
SBP (mmHg)	126.05 ± 10.81	129.45 ± 13.11	0.1239
DBP (mmHg)	78.83 ± 7.85	81.47 ± 9.87	0.1087
Height (cm)	167.04 ± 7.31	167.88 ± 8.19	0.5561
Weight (cm)	72.84 ± 12.33	74.86 ± 13.55	0.3980
BMI (kg/m^2^)	25.96 ± 3.27	26.33 ± 3.36	0.5479
Waist (cm)	93.43 ± 8.44	96.10 ± 9.31	0.1046
Hipline (cm)	99.41 ± 7.45	100.12 ± 8.66	0.6325
Waist-to-hip ratio	0.94 ± 0.05	0.96 ± 0.06	0.0491
Smoking history			
No smoking	40 (67.80)	41 (69.49)	0.8071
Occasional smoking	4 (6.78)	2 (3.39)	
Regular smoker	15 (25.42)	16 (27.12)	
Diabetes control			
Diet therapy			
No	22 (37.93)	28 (47.46)	0.2977
Yes	36 (62.07)	31 (52.54)	
Exercise therapy			
No	22 (37.93)	28 (47.46)	0.2977
Yes	36 (62.07)	31 (52.54)	
Nonalcoholic fatty liver control			
Diet therapy			
No	26 (47.27)	30 (50.85)	0.7572
Yes	29 (52.73)	29 (49.15)	
Exercise therapy			
No	30 (54.55)	31 (53.45)	0.9470
Yes	25 (45.45)	27 (46.55)	
Drug therapy			
No	51 (92.73)	50 (84.75)	0.6798
Yes	4 (7.27)	9 (15.25)	

Abbreviations: SBP: systolic blood pressure; DBP: diastolic blood pressure. Data are *n* (%) or mean (SD) ^∗^*P* < 0.05.

**Table 2:** Body weight, waist circumference, and liver biological indicators at different times.

Variable	Metformin				Pioglitazone + metformin			
	Baseline	12 weeks	24 weeks	Baseline -24 weeks	Baseline	12 weeks	24 weeks	Baseline -24 weeks
Primary endpoint								
*γ*-GT (IU/L)	41.35 ± 25.03	41.30 ± 31.13	40.55 ± 29.43	1.35 ± 20.47	46.39 ± 31.32	32.04 ± 21.75	30.99 ± 21.04^∗^	15.57 ± 24.92^∗∗^
C-peptide (mIU/L)	3.36 ± 3.47	1.29 ± 0.42	1.27 ± 0.24	1.94 ± 3.44	2.78 ± 1.60	1.28 ± 0.63	1.39 ± 0.83	1.43 ± 1.90
AST (IU/L)	28.05 ± 19.19	25.11 ± 13.08	23.66 ± 10.75	4.38 ± 14.75	26.50 ± 13.27	25.55 ± 14.37	21.58 ± 7.95	4.97 ± 11.42
ALT (IU/L)	37.23 ± 26.57	30.08 ± 15.90	31.10 ± 18.92	5.95 ± 19.26	37.37 ± 22.78	34.85 ± 23.14	26.37 ± 15.16	11.07 ± 19.22
FINS	12.05 ± 13.30	5.66 ± 2.04	5.80 ± 1.54	5.67 ± 11.81	10.46 ± 9.34	5.55 ± 1.01	5.40 ± 0.98	4.94 ± 8.99
Secondary endpoint								
LDL-C (mmol/L)	2.88 ± 0.83	2.73 ± 0.79	2.76 ± 0.77	0.10 ± 0.85	2.88 ± 1.46	2.79 ± 0.83	2.88 ± 0.87	0.06 ± 1.72
HDL-C (mmol/L)	1.31 ± 1.33	1.26 ± 0.33	1.21 ± 0.34	−0.09 ± 0.27	1.22 ± 0.50	1.28 ± 0.30	1.25 ± 0.28	−0.03 ± 0.57
TC (mmol/L)	3.41 ± 6.75	2.13 ± 1.40	2.21 ± 1.53	1.39 ± 7.31	2.44 ± 2.34	1.97 ± 1.57	1.80 ± 0.96	0.57 ± 2.44
TG (mmol/L)	4.87 ± 0.86	4.64 ± 1.05	5.64 ± 5.58	0.80 ± 5.65	4.66 ± 0.96	4.71 ± 0.98	4.79 ± 1.00	0.09 ± 0.88
Weight (kg)	74.86 ± 13.55	74.60 ± 12.97	75.05 ± 13.27	0.08 ± 4.85	72.84 ± 12.33	71.44 ± 11.76	72.58 ± 11.29	−0.15 ± 3.91
Waist circumference (cm)	96.10 ± 9.31	95.3 0 ± 9.01	95.86 ± 9.24	−0.36 ± 3.84	93.43 ± 8.44	92.50 ± 7.92	95.33 ± 16.27	2.60 ± 14.79
HbA1c (%)	8.25 ± 1.02	6.50 ± 0.58	6.53 ± 0.71	1.77 ± 1.18	8.13 ± 0.93	6.35 ± 0.86	6.48 ± 1.11	1.66 ± 1.17
FBG (mmol/L)	8.62 ± 2.16	6.78 ± 1.19	7.07 ± 2.42	1.77 ± 2.41	8.21 ± 1.85	6.79 ± 2.80	6.78 ± 1.84	1.31 ± 3.06
PBG (mmol/L)	17.57 ± 21.66	13.89 ± 14.24	10.81 ± 2.60	7.55 ± 24.43	14.07 ± 3.49	10.20 ± 3.34	9.98 ± 3.86	4.09 ± 4.32
IR index	4.12 ± 3.75	1.75 ± 0.92	1.89 ± 0.82	2.53 ± 4.11	3.60 ± 3.11	1.74 ± 1.04	1.60 ± 0.59	2.16 ± 3.19
POINS2H (mIU/L)	2.29 ± 0.86	4.41 ± 15.02	2.48 ± 1.25	0.26 ± 1.12	2.27 ± 1.13	2.15 ± 1.22	2.30 ± 1.95	0.14 ± 2.11

Note: data are mean ± standard deviation. ^∗^*P* < 0.05 and ^∗∗^*P* < 0.01 compared with the metformin group. Abbreviation: ALT: alanine aminotransferase; AST: aspartate aminotransferase; TG: triglycerides; TC: total cholesterol; HBA1C: glycated hemoglobin; LDL: low-density lipoprotein; HDL: high-density lipoprotein; HBA1C: glycated hemoglobin; POINS2H: postprandial insulin; FBG: fasting blood glucose; FINS: fasting insulin; PBG: 2-hour postprandial blood glucose.

**Table 3:** Incidence of adverse events in each group.

Adverse events	Metformin		Pioglitazone + metformin		Fisher *P*
	Frequency	Rate	Frequency	Rate	
Total adverse events	8	13%	7	8.33%	0.5585
Adverse events related to study	6	10.00%	5	6.67%	0.743
Adverse events not related to study	3	5.00%	2	3.33%	1
Serious adverse events	1	1.67%	1	1.67%	1

^∗^ *P* < 0.05.

**Table 4:** Drug-related adverse events in each group.

Adverse events	Metformin		Pioglitazone + metformin	
	Frequency	Rate	Frequency	Rate
Diarrhea	10	100%	3	60%
Urinary occult blood	0	0	1	20%
Cardiovascular abnormalities	0	0	1	20%
Total	10		5	

## Data Availability

The original contributions presented in the study are included in the article. Further inquiries can be directed to the corresponding author.
